# Identifying genes with tri-modal association with survival and tumor grade in cancer patients

**DOI:** 10.1186/s12859-018-2582-7

**Published:** 2019-01-08

**Authors:** Minzhe Zhang, Tao Wang, Rosa Sirianni, Philip W. Shaul, Yang Xie

**Affiliations:** 1 Department of Clinical Sciences, Quantitative Biomedical Research Center, University of Texas Southwestern Medical Center, 5323 Harry Hines Blvd, Dallas, TX 75390 USA; 2 Harold C. Simmons Comprehensive Cancer Center, University of Texas Southwestern Medical Center, 5323 Harry Hines Blvd, Dallas, TX 75390 USA; 3 Center for the Genetics of Host Defense, University of Texas Southwestern Medical Center, 5323 Harry Hines Blvd, Dallas, TX 75390 USA; 4 Department of Pediatrics, Division of Pulmonary and Vascular Biology, University of Texas Southwestern Medical Center, 5323 Harry Hines Blvd, Dallas, TX 75390 USA; 5 Department of Bioinformatics, University of Texas Southwestern Medical Center, 5323 Harry Hines Blvd, Dallas, TX 75390 USA

**Keywords:** Expectation maximization, Oncogene, Tumor suppressor gene, Survival, Breast Cancer

## Abstract

**Background:**

Previous cancer genomics studies focused on searching for novel oncogenes and tumor suppressor genes whose abundance is positively or negatively correlated with end-point observation, such as survival or tumor grade. This approach may potentially miss some truly functional genes if both its low and high modes have associations with end-point observation. Such genes act as both oncogenes and tumor suppressor genes, a scenario that is unlikely but theoretically possible.

**Results:**

We invented an Expectation-Maximization (EM) algorithm to divide patients into low-, middle- and high-expressing groups according to the expression level of a certain gene in both tumor and normal patients. We found one gene, ORMDL3, whose low and high modes were both associated with worse survival and higher tumor grade in breast cancer patients in multiple patient cohorts. We speculate that its tumor suppressor gene role may be real, while its high expression correlating with worse end-point outcome is probably due to the passenger event of the nearby ERBB2’s amplification.

**Conclusions:**

The proposed EM algorithm can effectively detect genes having tri-modal distributed expression in patient groups compared to normal genes, thus rendering a new perspective on dissecting the association between genomic features and end-point observations. Our analysis of breast cancer datasets suggest that the gene ORMDL3 may have an unexploited tumor suppressive function.

**Electronic supplementary material:**

The online version of this article (10.1186/s12859-018-2582-7) contains supplementary material, which is available to authorized users.

## Background

Alterations in oncogenes or tumor suppressor genes underlie the driving forces of carcinogenesis. An oncogene is a gene that causes cancer through activating mutation or expression at high levels, while for a tumor suppressor gene, it is the loss or reduction of function that leads to cancer. Research in cancer biology has identified hundreds of genes involved in different stages of tumorigenesis [7, 17]. The alterations in these oncogenes or tumor suppressor genes can come from a variety of sources, such as single nucleotide polymorphisms (SNPs), copy number variations (CNV), chromosomal regions, viral integration, gene fusions, etc. There is another type of event called a passenger mutation, which also commonly occurs in tumor tissues. However, such passenger mutations have no effect on the growth of tumors and they usually hitchhike on a near-by tumor driver gene’s alteration. It is an important research question to distinguish true tumor driver mutations from artefact events such as passenger mutations in order to better elucidate tumor oncogenesis and evolution. As the names “oncogene” and “tumor suppressor gene” suggest, previous systematic searches for tumor driver genes have mostly adopted the paradigm that a positive association between up-regulation and gain of function vs. tumor proliferation and worse survival hints at a possible oncogene, while for tumor suppressor genes, a negative association is expected. For example, Bric et al. conducted an RNA interference (RNAi) screen for tumor suppressors through selecting for small hairpin RNAs (shRNAs) capable of accelerating lymphomagenesis in a mouse model [4]. Koso et al. mobilized the Sleeping Beauty transposon system in mice and profiled insertions that promoted medulloblastoma formation in the cerebellum [15]. Wrzeszczynski et al. carried out a bioinformatics screen for candidate ovarian cancer oncogenes or tumor suppressors by first looking for genes with significant amplification or deletion across tumor samples [31]. Regardless of the different specific designs, there is one common feature shared by most such screening studies. They all assume a monotone (either positive or negative) relationship between the end-point outcome and their genes of interest.

However, there remains the possibility that a true driver gene could actually exhibit a non-linear association with end-point observations. That is to say, both its up-regulation end and down-regulation can lead to aggressive tumor growth or metastasis, or vice versa. With a slight abuse of terms, “regulation” here includes any type of copy number variation, mutation, or RNA expression level change. Recently, Shen et al. explored the existence of such genes, which can potentially perform both oncogenic and tumor suppressive functions, through database searching and text mining [24]. They identified 83 genes that have dual functional annotation according to the literature. Most of these genes are transcription factors. They can both positively and negatively regulate transcription, which serves as the basis for their potential dual role in cancer development. These genes usually carry genomic mutation patterns similar to those of oncogenes, and expression patterns resembling those of tumor suppressor genes. TP53 is an example of one whose tumor suppressive effect, as exerted by activating DNA repair proteins, arresting the cell cycle and initiating apoptosis, is well known. On the other hand, more than 80% of the somatic and germline TP53 alterations found are missense mutations rather than nonsense or frame-shift mutations, which usually lead to loss of function. The strong selection to maintain expression of the full-length p53 mutant protein and its accumulation in the nucleus is an implication of gain-of-function and oncogenic mutation [26]. An in vivo knock in experiment has shown that many mutant p53 variants are essential for neoplastic transformation [29]. Another close example is Notch, which is an oncogene in cancer types like T cell acute lymphoblastic leukemia (ALL), and a tumor suppressor gene in other types like B cell ALL [18]. A more concrete example would be c-Myc whose dual role in leukemia was described by Uribesalgo et al. [30]. They showed that the c-Myc/RARα complex could function either as an activator or a repressor based on the c-Myc phosphorylation status.

Although to the extent of our knowledge at present, there is no solid evidence of a gene that can perform both oncogenic and tumor suppressive effects in one cell line, the possibility cannot be ruled out. Such genes may be overlooked by traditional approaches, as these assume a linear association. Even if not a true bifunctional gene, a gene bearing a true function and a passenger event (e.g. a tumor suppressor gene coincidentally amplified with a nearby oncogene) can easily confound analysis, leading to its failure to be discovered as a hit. Therefore, it is important and worthwhile to explore whether there exists a non-linear association between genomic features and end-point outcomes, what the abundance is, and how it occurs if it does exist. As far as we know, no such study has been proposed to answer these questions.

In this study, we carried out a large-scale bioinformatics screen with the motivation to search for genes that have tri-modal association with end-point observations. First, we divided patients or cell lines into “lower than normal” (“low”), “similar to normal” (“middle”) and “higher than normal” (“high”) groups based on the expression levels of each investigated gene in tumor samples with respect to normal samples. To do this, we devised an algorithm based on Expectation-Maximization (EM) [9] that takes into consideration the expression levels of both normal samples and tumor samples for each gene. Then we focused on a specific scenario where candidate targets whose “low” and “high” groups of patients were both associated with worse survival and higher tumor grade compared to the “middle” group of patients. We termed this a “tri-modal” association.

This study will mainly focus on breast cancer, which is the most common type of invasive cancer in women. Breast tumors can be graded with the Nottingham Histologic Score system [25]. In this system, a grade of 1, 2 or 3 is given to a breast tumor, where 3 has the poorest chance of prognostic survival. A number of tumor driver genes have been previously identified in breast cancers. For example, ERBB2, ESR1 and c-myc are breast tumor oncogenes; p53, p27, Skp2, BRCA-1 and BRCA-2 are breast tumor suppressors [20, 32]. Breast cancer can be divided into 5 subtypes according to the PAM50 assay [21], which include luminal A, luminal B, HER2-enriched, basal-like, and normal-like subtypes. The basal-like breast tumor subtype largely overlaps the triple negative type of breast cancer, which lacks or shows a low level of ESR1 and PGR expressions, and lacks ERBB2 amplification. Estrogen-receptor (ER) negative breast cancer, which generally includes basal and HER2 subtypes, is characterized by aggressive clinical behavior and resistance to hormone deprivation therapy [28]. In our study, we replicated our analysis across an array of breast tumor patient cohorts, including the following: (1) the Metabric study [8], where a total of ~ 2000 patients are available and divided into a discovery set and a validation set; (2) the Cancer Genome Atlas (TCGA) [5] breast cancer study, where ~ 1000 patients are available; (3) the GSE18229 study [22], where 337 breast cancer patients are available; (4) the GSE20624 study [1], where 344 breast cancer patients are available; (5) the GSE20685 study [14], where 327 breast cancer patients are available; and (6) the GSE22133 study [12, 13], where 359 breast cancer patients are available.

## Results

### Grouping of patients into 3 modes by EM algorithm

**Fig. 1:**
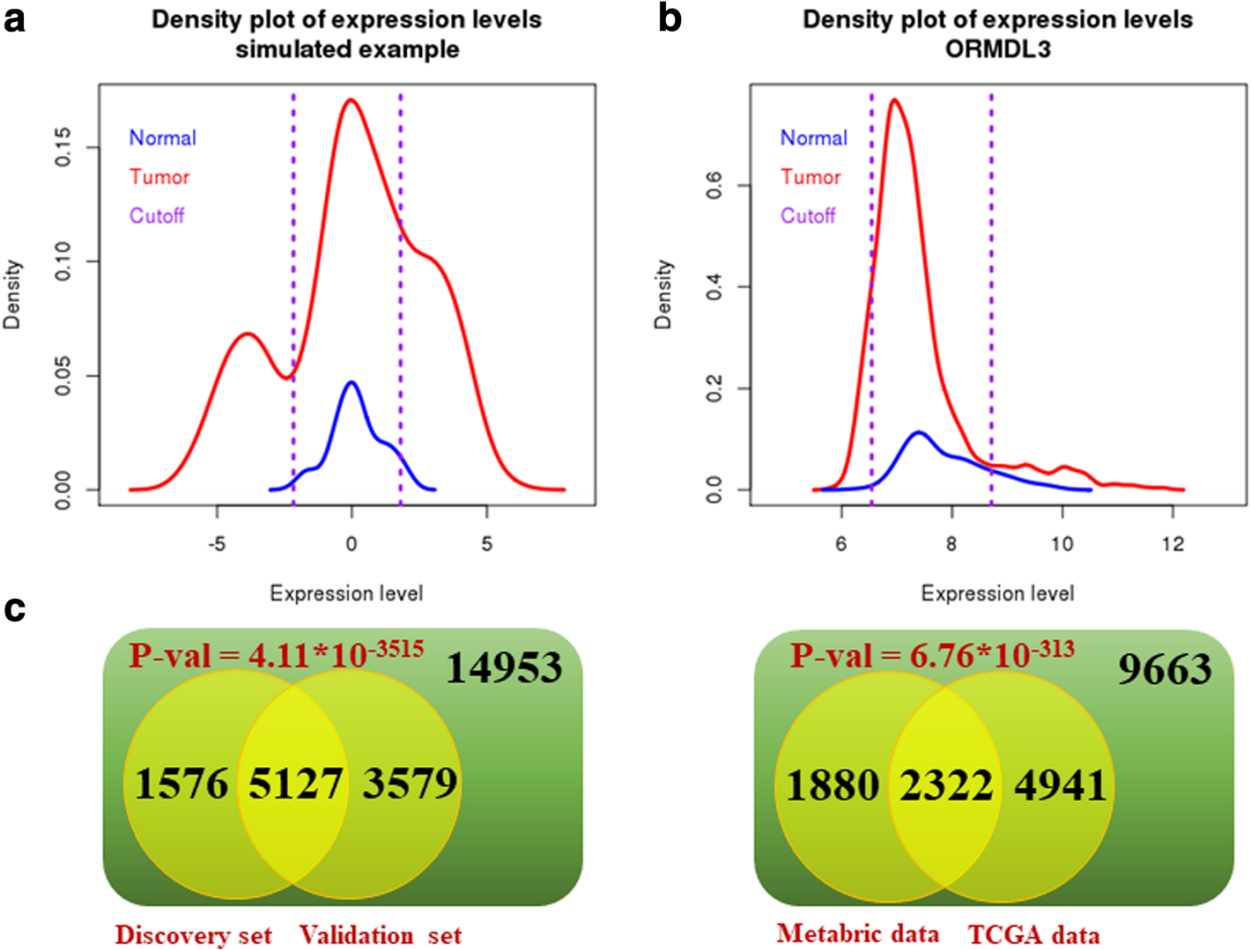
Applied EM algorithm to discover trimodal genes. **a** A simulated example to verify the validity of the EM algorithm. **b** The distribution of the expression values for the gene ORMDL3 in the Metabric discovery set. **c** The common genes found to have trimodal distribution between the Metabric discovery set vs. Metabric validation set, and between the Metabric data and TCGA data. Hypergeometric p is given to show the significance of overlap of trimodality or non-trimodality across different cohorts of patients

We focused on the cases where the tumor patients can be grouped into “low”, “middle” and “high” groups according to expression of a certain gene. The “middle” group should have expression levels similar to normal patients, while both “low” and “high” groups should have worse survival and higher tumor grades than “middle” group patients. This scenario enables a natural explanation that the “low” and “high” groups of patients suffer from a cancerous condition that deviated from the “middle” and normal patients, and the expression of this gene may be the cause for this cancerous condition. We devised an EM algorithm for this task. To test that the EM algorithm was working properly, we simulated the tumor population as a mixture of Gaussian (− 4,1), Gaussian (0,1) and Gaussian (3,1) with numbers of samples equal to 100, 250 and 150. We also simulated the normal population as Gaussian (0,1) with number of samples equal to 50. The EM algorithm detected the mean vector to be (− 3.92, − 0.076, 2.93), mixing proportion to be (0.21, 0.59, 0.29) and the standard deviation to be 1.006, which are very close to the true parameters (Fig. 1a). We used the Metabric data as our primary dataset, where we perform the EM algorithm on discovery set against the normal set, and the validation set against the normal set, respectively. For example, Fig. 1b shows the distribution of the expression values for the gene ORMDL3 in the discovery set. The distribution of ORMDL3 in the validation set was very similar (Additional file 1: Figure S1). This screen was conducted on all 25235 genes available in the expression data and returned 6703 and 8706 genes with tri-modal distribution in the discovery set and validation set, respectively. The degree of trimodality varies greatly from weak to strong for these genes. In Fig. 1c, we showed the overlap between these two lists of genes. We also performed the trimodality search on the TCGA BRCA breast cancer patients. Figure 1c also shows the overlap between the common trimodal genes found in the Metabric dataset and the trimodal genes found in the TCGA dataset, comparing only genes that were available in both datasets. The hypergeometric *p* values show that genes tended to consistently show trimodality or non-trimodality across different cohorts of patients.

### Identify genes with tri-modal association with prognostic survival and tumor grade

**Fig. 2:**
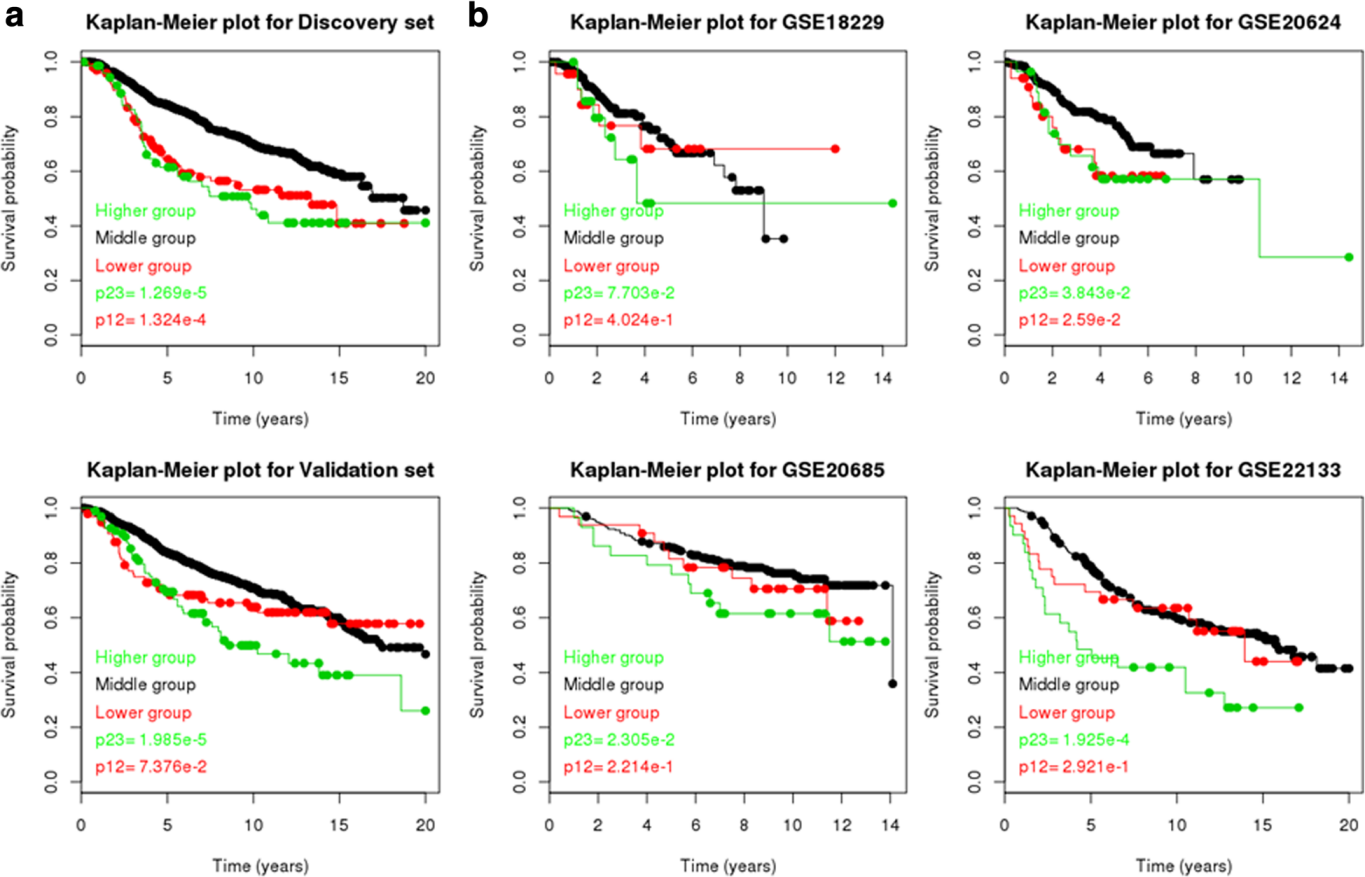
Association of ORMDL3’s “low”, “middle” and “high” modes with prognostic survival. Survival data is regressed on the categorical variable encoding these modes. P12 is the *p* value of testing whether “low” mode patients have worse survival than “middle” mode patients. P23 is the p value of testing whether “high” mode patients have worse survival than “middle” mode patients. **a** Metabric discovery set and validation set. **b** GSE18229, GSE20624, GSE20685 and GSE22133 datasets

Using each gene that had a trimodal distribution and each mode whose proportion was at least 5% within both the Metabric discovery set and Metabric validation set, we tried to investigate whether both the “high” and “low” mode correlated significantly (*p* < 0.05) with worse prognostic survival and higher tumor grade than the “middle” mode. No gene satisfies this criterion, but one gene, ORMDL3, was very close (Fig. 2a and Table 1). The EM algorithm detected 10.0 and 7.7% of all discovery set patients to be in the “low” and “high” modes; and 10.0 and 9.9% of all validation set patients to be in the “low” and “high” modes. To test if this observation was robust, we tried to replicate the analysis in the TCGA BRCA cohort and 4 smaller cohorts, including GSE18229, GSE20624, GSE20685, and GSE22133. In these four smaller cohorts, there were no normal patients to conduct the EM algorithm. Therefore, we took the average of the proportions found in the Metabric cohorts and split each cohort into 10.0, 81.1 and 8.8% according to the expression levels of ORMDL3. Figure 2b shows the results of the survival analysis. It can be seen that the trimodal association between ORMDL3 and prognostic survival was significant (p12 <  0.05 and p23 < 0.05) for GSE20624. This relationship was non-significant for GSE18229, GSE20685 and GSE22133, but at least the trimodal trend was correct (p12 < 0.5 and p23 < 0.5). Table 1 shows the association between ORMDL3 expression and tumor grade. It can be seen that patients whose ORMDL3 expression fell into the low mode always had a significantly (*p* < 0.05) higher grade than those whose ORMDL3 expression fell into the middle mode. Patients whose ORMDL3 expression fell into the high mode didn’t always have significantly (*p* < 0.05) higher grades than those whose ORMDL3 expression fell into the middle mode, but the trend was still correct (*p* < 0.5) in most cases.

**Table 1:** Association of ORDML3 trimodal expression with tumor grade

Data set	Patient number						*P*-value	
	Expression			Tumor grade			“Low” vs. “Middle”	“High” vs. “Middle”
	low	middle	high	Stage 1	Stage 2	Stage 3		
Metabric discovery	100	820	77	72	415	510	9.0 × 10^−7^	4.6 × 10^− 10^
Metabric validation	94	719	92	98	360	447	1.1 × 10^−5^	2.9 × 10^−8^
GSE18229	10	186	27	24	74	125	2.7 × 10^−3^	3.9 × 10^−1^
GSE20624	34	253	29	19	97	200	3.9 × 10^−2^	7.8 × 10^− 1^
GSE22133	25	187	20	26	100	106	1.7 × 10^−3^	5.4 × 10^−2^

Tailed p value is for the null hypothesis that “low” (“high”) group patients tend to have lower grade tumors when compared to “middle” group patients. GSE20685 does not have tumor grade data, so the p value is not calculated

### The phenotype of ORMDL3 amplification may be artefact of nearby ERBB2 expression

**Fig. 3:**
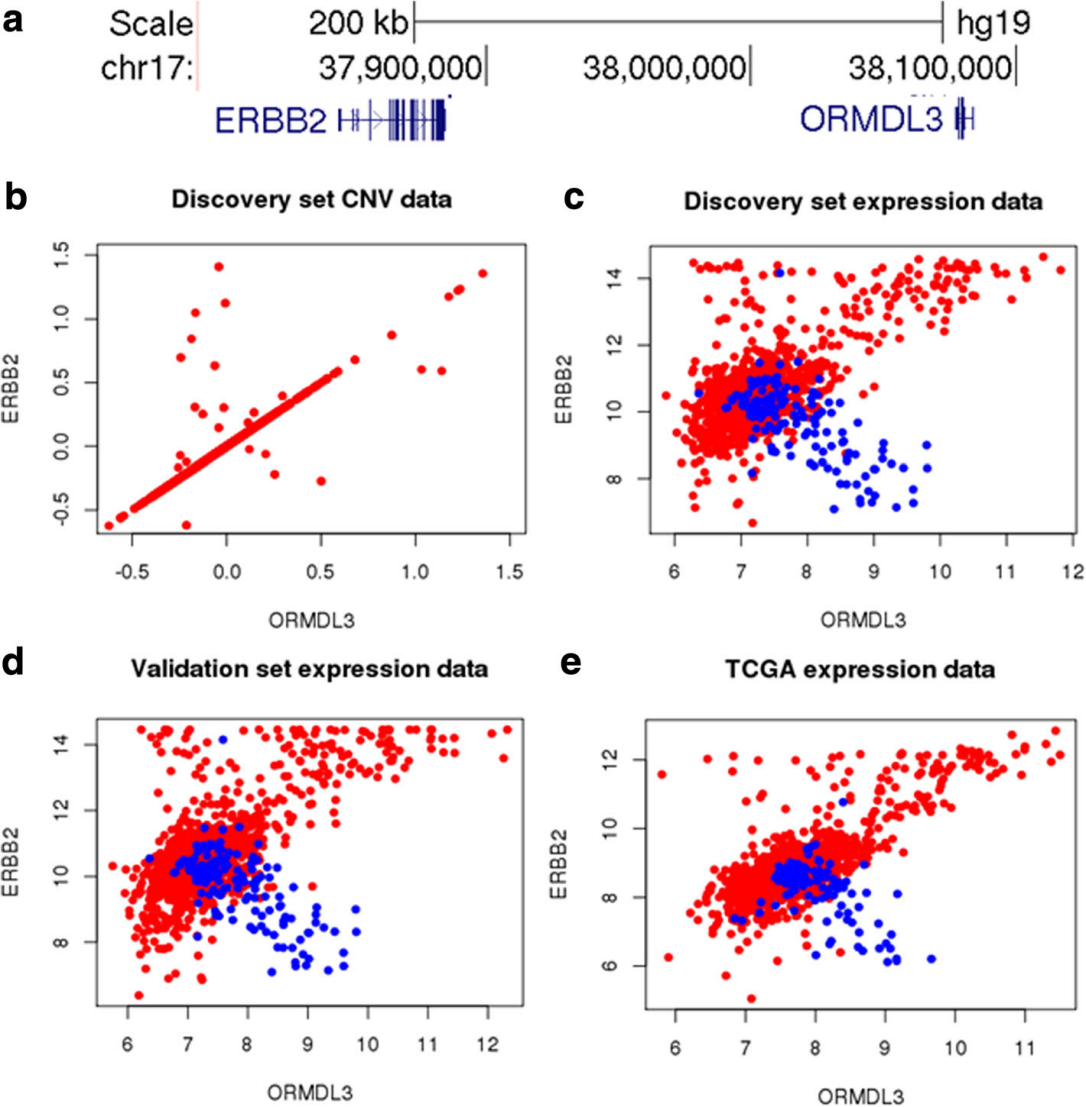
The phenotype of ORMDL3 amplification may be an artefact of nearby ERBB2 expression. **a** Genome Browser visualization of ORMDL3 and ERBB2. **b** Copy Number Variations of ORMDL3 and ERBB2 for the Metabric discovery set patients (**c–e**) RNA expression levels of ORMDL3 and ERBB2 for the Metabric discovery set, Metabric validation set, and TCGA BRCA dataset. Blue dots represent normal samples and red dots represent tumor samples

Overall, we conclude that both the up-regulation and down-regulation of ORMDL3 were correlated with bad prognosis and higher tumor grade in breast cancer patients, although this observation did not reach statistical significance in some small validation datasets. We then asked whether ORMDL3 was the driving factor for both the up-regulation phenotype and down-regulation phenotype. We noticed that ORMDL3 is only about 200 kb away from ERBB2/HER2 (Fig. 3a), which is a well-known tumor driver in multiple cancers, including breast cancer [11]. 15–25% of breast tumors carry a high-level amplification of ERBB2 [10], and ERBB2-overexpressing in breast cancer leads to substantially lower overall survival rates [27].

We hypothesized that the phenotype of up-regulation of ORMDL3 is a passenger event of nearby ERBB2’s amplification. Indeed, when we plotted the Copy Number Variations of ORMDL3 and ERBB2 for the Metabric discovery set patients in Fig. 3b, we could see that ORMDL3 and ERBB2 were often amplified or deleted together. When ORMDL3 was amplified, ERBB2 was always amplified, but not vice versa. This could be replicated in the Metabric validation dataset and TCGA BRCA dataset (Additional file 1: Figure S2). Consistent with CNV data, the ORMDL3 and ERBB2 expression levels were positively correlated for the tumor samples, but with a significant portion of outliers in the upper-left corner (Fig. 3c-e). Interestingly, in normal samples, ORMDL3 and ERBB2 were negatively correlated in all three datasets examined. In addition, tumor and normal samples tended to occupy different regions in the ORMDL3-by-ERBB2 graphs.

Moreover, we calculated the relationship between gene essentiality vs. gene expression. For ORMDL3 (Additional file 1: Figure S3a), expression has a slightly positive association with gene essentiality. But for an oncogene, the higher it is expressed, the more likely the tumor cell line is reliant on this gene’s expression for survival. In turn, this cell line is more sensitive to knockdown of the oncogene, leading to a more negative gene essentiality score. Indeed, the expression-by-essentiality plots show strong negative associations for some oncogenes (Additional file 1: Figure S3b-e), but not for tumor suppressors (Additional file 1: Figure S3f-k) [6, 16]. Although inconclusive, this analysis suggests that ORMDL3 has no oncogenic effect.

### ORMDL3 may be a breast tumor suppressor

Based on the above-mentioned evidence, it is reasonable to suspect that the up-regulation of ORMDL3 is merely a passenger event of ERBB2 amplification. However, we hypothesized that the association between down-regulation of ORMDL3 and worse survival prognosis as well as higher tumor grade is due to the possible tumor suppressor effect of ORMDL3. To investigate this hypothesis, we conducted a multivariable analysis incorporating the 3 modes of ORMDL3 expression together with other variables for the Metabric discovery set survival data (Table 2). These variables include the expression level of ERBB2 as well as many other clinical variables. According to the table, the association of the up-regulation of ORMDL3 with worse survival is no longer significant (*p* = 0.72), while the down-regulation of ORMDL3 with worse survival is still significant (*p* = 0.002) after adjustment. We also extended this analysis to the other datasets, though not all of them fully captured these biological and clinical variables. So in this analysis, we conducted multivariable regression of the 3 modes of ORMDL3 expression only with ERBB2 for both survival and tumor grade data (Additional file 1: Table S1). We can see that the *p* values representing the down-regulation of ORMDL3 did not change too much from the univariate p values, while p values representing the up-regulation of ORMDL3 are mostly much less significant than the univariate p values. These results again confirmed our speculation that up-regulation of ORMDL3 is an artefact while ORMDL3 may be a new tumor suppressor.

**Table 2:** Multivariable survival analysis with ORMDL trimodal expression and other variables

Variables	coefficient	p-value
ORMDL expression (“low” vs. “middle”)	0.513	0.002
ORMDL expression (“high” vs. “middle”)	−0.140	0.72
ERBB2 expression	0.182	0.001
ESR1 expression	−0.063	0.87
PGR expression	−0.175	0.99
Pam50subtype – Her2	−0.206	0.78
Pam50subtype – LumAB	−0.273	0.81
Pam50subtype – Normal	0.077	0.40
Age at diagnosis	0.148	0.002
Stage	0.024	0.36
Lymph nodes positive	0.110	< 0.001

Analysis was done in Metabric discovery set

## Discussion

ORMDL3 is an endoplasmic reticulum-located transmembrane protein. It is mainly known as a negative regulator of sphingolipid synthesis [3], and it is involved in asthma as well as a series of autoimmune disorders [23]. However, currently few research papers have demonstrated whether it is involved in cancer. To validate its hypothetic role as a tumor suppressor, further experimental validation would need to be carried out. Similar analysis can also be carried out in the future in other cancer datasets to identify potential functional genes in cancer that may be missed by traditional studies.

## Conclusions

In this study, we proposed an EM model to detect genes with trimodal expression in cancer patients to answer our specific question of interest: can a gene be both an oncogene and a tumor suppressor in a certain scenario? Applying our EM algorithm to the Metabric breast cancer dataset, we identified the gene ORMDL3, whose low and high expression are both associated with higher tumor grade and worse survival outcome. Down-stream analysis suggests the oncogenic effect of ORMDL3 may be an artefact by its nearby oncogene ERBB2 amplification, while its tumor suppressor role cannot be ruled out. Current research into ORMDL3 is focused on asthma and autoimmune diseases, so the functional study of its role in cancer is still blank. Future bench work is needed to validate its tumor suppressive effect in breast cancer. Taken together, this study provides a novel angle to look for oncogenes and tumor suppressors, linking trimodal gene abundance to endpoint observation.

## Methods

### Curation of breast cancer studies

The Metabric study datasets were downloaded from EMBL-EBI with the study ID EGAS00000000083. Study datasets were comprised of the discovery set and the validation set, as well as a third smaller group of normal control samples. For the expression data of each set of samples, probe-level data were aggregated to the gene level and each sample was adjusted using quantile normalization. For the copy number variation variant data, each gene’s CNV status was found by calculating the mean of the values of the probes covering that gene. The TCGA Breast invasive carcinoma (BRCA) study data were also downloaded and contained mostly tumor samples and some normal samples. The HiSeq expression data were log transformed and median centered. The BRCA CNV data were downloaded from Firehose, and GISTIC gene-level output were used directly. For the GSE18229 study and the GSE20624 study, expression data were downloaded from the UNC microarray database, aggregated from the probe-level to the gene-level and quantile normalized. For the GSE20685 study, the expression data were downloaded from the GEO database. For the GSE22133 study, the expression data were aggregated from the probe level to the gene level and quantile normalized. For the CNV data, the values of the probes covering each gene were averaged to become the CNV status of that gene.

### EM algorithm

We devised an EM algorithm to separate the whole tumor patient population into 3 groups, “higher than normal”, “similar to normal” and “lower than normal”. To do this, we assumed that the expression values of a certain gene in the tumor patient population were a mixture of 3 Gaussian distributions (3 modes), corresponding to each of the 3 groups mentioned above. We assumed those of the normal patient corresponded only to the middle component. To avoid assignment of a patient to an unreasonable mode, we assumed these 3 Gaussian distribution shared the same variance. Then the log likelihood function could be written as:

$$ LL\left({\overrightarrow{\mathrm{x}}}_{tumor},{\overrightarrow{\mathrm{x}}}_{normal};\overrightarrow{\pi},\overrightarrow{\mu},\sigma \right)=\sum \limits_{i=1}^{\# tumor}\log \left(\sum \limits_{j=1}^3f\left({\mathrm{x}}_{\mathrm{tumor},i};{\mu}_j,\sigma \right)\times {\pi}_j\right)+\sum \limits_{i=1}^{\# normal}\log \left(f\left({\mathrm{x}}_{normal,i};{\mu}_2,\sigma \right)\right) $$

$f\left(\mathrm{x};\mu, \sigma \right)=\kern0.5em \frac{1}{\sqrt{2\pi}\sigma }{e}^{-\frac{{\left(\mathrm{x}-\mu \right)}^2}{2{\sigma}^2}}$ is the density function of normal distribution. ${\overrightarrow{x}}_{tumor}$ and ${\overrightarrow{x}}_{normal}$ are the vectors of expression levels of a certain gene in the tumor patient population and normal patient population. $\overrightarrow{\pi}$ is a 3-element vector specifying the proportion of patients that belong to each of the 3 modes. $\overrightarrow{\mu}$ is a 3-element vector specifying the mean of the 3 Gaussian distributions, subject to *μ*_1_ ≤ *μ*_2_, *μ*_2_ ≤ *μ*_3_. *σ* is the standard deviation of the 3 Gaussian distributions.

For each round, the EM algorithm was started by updating the responsibilities $\overrightarrow{\gamma}$, which is a vector with #*tumor* elements: ${\gamma}_{i,j}=\frac{f\left({\mathrm{x}}_{tumor,i};{\mu}_j,\sigma \right)}{\sum \limits_{k=1}^3f\left({\mathrm{x}}_{tumor,i};{\mu}_k,\sigma \right)}$. Then $\overrightarrow{\pi}$ is updated by ${\pi}_j=\frac{\sum \limits_{i=1}^{\# tumor}{\gamma}_{i,j}}{\# tumor}$ , $\overrightarrow{\mu}$ is updated by ${\mu}_j=\frac{\sum \limits_{i=1}^{\# tumor}{x}_{tumor,i}{\gamma}_{i,j}+I\left(j=2\right)\sum \limits_{i=1}^{\# normal}{x}_{normal,i}}{\sum \limits_{i=1}^{\# tumor}{\gamma}_{i,j}+I\left(j=2\right)\times \# normal}\left(j=1,2,3\right)$, but the inequality bounds require that:

if *μ*_1_ > *μ*_2_, *μ*_2_ ≤ *μ*_3_, then $$ {\mu}_1={\mu}_2=\frac{\sum \limits_{j=1}^2\sum \limits_{i=1}^{\# tumor}{x}_{tumor,i}{\gamma}_{i,j}+I\left(j=2\right)\sum \limits_{i=1}^{\# normal}{x}_{normal,i}}{\sum \limits_{j=1}^2\sum \limits_{i=1}^{\# tumor}{\gamma}_{i,j}+I\left(j=2\right)\times \# normal} $$;

if *μ*_1_ ≤ *μ*_2_, *μ*_2_ > *μ*_3_, then $$ {\mu}_2={\mu}_3=\frac{\sum \limits_{j=2}^3\sum \limits_{i=1}^{\# tumor}{x}_{tumor,i}{\gamma}_{i,j}+I\left(j=2\right)\sum \limits_{i=1}^{\# normal}{x}_{normal,i}}{\sum \limits_{j=2}^3\sum \limits_{i=1}^{\# tumor}{\gamma}_{i,j}+I\left(j=2\right)\times \# normal} $$;

and if *μ*_1_ > *μ*_2_, *μ*_2_ > *μ*_3_, then ${\mu}_1={\mu}_2={\mu}_3=\frac{\sum \limits_{j=1}^3\sum \limits_{i=1}^{\# tumor}{x}_{tumor,i}{\gamma}_{i,j}+I\left(j=2\right)\sum \limits_{i=1}^{\# normal}{x}_{normal,i}}{\sum \limits_{j=1}^3\sum \limits_{i=1}^{\# tumor}{\gamma}_{i,j}+I\left(j=2\right)\times \# normal}$.

Finally, *σ* is updated by $$ {\left[\frac{\sum \limits_{i=1}^{\# tumor}\sum \limits_{j=1}^3{\gamma}_{i,j}{\left({\mathrm{x}}_{tumor,i}-{\mu}_j\right)}^2+\sum \limits_{i=1}^{\# normal}{\left({\mathrm{x}}_{normal,i}-{\mu}_2\right)}^2}{\sum \limits_{i=1}^{\# tumor}\sum \limits_{j=1}^3{\gamma}_{i,j}+\# normal}\right]}^{\raisebox{1ex}{$1$}\!\left/ \!\raisebox{-1ex}{$2$}\right.} $$.

The EM iterations were stopped when the log likelihood reached convergence. When *μ*_1_ < *μ*_2_, *μ*_2_ < *μ*_3_, and *π*_*i*_ > 0.01, *i* = 1, 2, 3 were all satisfied, this gene was said to exhibit trimodality distribution. Then two cutoff values were calculated by ${cutoff}_{12}=\frac{\mu_1^2-{\mu}_2^2-2{\sigma}^2\log \left(\frac{\pi_1}{\pi_2}\right)}{2\left({\mu}_1-{\mu}_2\right)}$ and ${cutoff}_{23}=\frac{\mu_2^2-{\mu}_3^2-2{\sigma}^2\log \left(\frac{\pi_2}{\pi_3}\right)}{2\left({\mu}_2-{\mu}_3\right)}$. Sometimes *cutoff*_12_ > *μ*_2_ or *cutoff*_12_ < *μ*_1_ could occur. When that happened, an ad hoc rule applied to set *cutoff*_12_ at the 10% quantile of the expression values of the tumor samples. Similarly, *cutoff*_23_ was set at the 90% quantile when *cutoff*_23_ > *μ*_3_ or *cutoff*_23_ < *μ*_2_. Finally the true membership of each tumor sample to the three modes was decided by comparing their expression values to *cutoff*_12_ and *cutoff*_23_. An empirical *π* was calculated by the proportion of tumor patients belonging to each mode.

### Gene essentiality analysis

The gene essentiality screening data were downloaded from the 2012 Cancer Discovery study [19]. In this study, a continuous GARP score was defined for each gene in every cell line. A lower score for a gene meant that the cell line was more reliant on the expression of this gene for survival. We used the expression data downloaded from the Cancer Cell Encyclopedia (CCLE) website [2]. The whole CCLE dataset contained the expression data of 58 breast cancer cell lines. 29 of these cell lines were also used in the gene essentiality screening study.

### Statistical tests

Survival analysis performed in this study was done using functions from the R survival package. To test the tri-modal association of each gene’s expression level with overall survival, the “low”, “middle”, and “high” categorical variables were input into the Cox proportional hazard model, with or without adjusting for other variables. The *P* value for the “low” group was assigned by testing the null hypothesis that “low” group patients had no worse overall survival than “middle” group patients, and the same applied for “high” group *p* values. All survival analysis was censored at 20 years.

To test the proportional trend of two groups of patients in tumor graded 1, 2 and 3, a modified version of prop.trend.test function from the stats R package was used. The p value generated by prop.trend.test was from a two-tailed test, while a one-tailed p value was calculated from it by examining the sign of the coefficient. The one-tailed p value was for the null hypothesis that “low” (“high”) group patients tended to have lower grade tumors when compared to “middle” group patients. To compare “low” vs. “middle” groups for example, the test in essence generated a smaller p value when more advanced grade tumors were more likely to be “low” group patients rather than “middle” group patients.

## Additional file

Additional file 1: **Figure S1.** The distribution of the expression values for the gene ORMDL3 in the Metabric validation set. **Figure S2.** The distribution of the expression values for the gene ORMDL3 in the Metabric validation set and TCGA BRCA patients. **Figure S3.** Scatterplots of gene expression levels vs. gene essentiality scores (GARP scores). Yellow dots are the breast cancer cells that exists in both CCLE and the shRNA screening data. The expression values and GARP scores are all adjusted by breast cancer subtypes. The purple curve is fitted by linear regression. (a) ORMDL3 (b-e) breast cancer oncogenes (f-k) breast tumor suppressors. **Table S1.** Multivariable survival analysis with ORMDL trimodal expression and ERBB2 expression. (DOCX 483 kb)

## Abbreviations

ALL: Acute lymphoblastic leukemia
CCLE: Cancer Cell Encyclopedia
CCLE: Copy number variations
EM: Expectation-Maximization
RNAi: RNA interference
shRNA: Small hairpin RNA
SNP: Single nucleotide polymorphism
