# Supplementary material for: Identifying genes with tri-modal association with survival and tumor grade in cancer patients
Source: BMC Bioinformatics. 2019 Jan 8;20:13. doi: 10.1186/s12859-018-2582-7 (PMC6323748; doi:10.1186/s12859-018-2582-7)
Supplement: Supplementary file 1 — Figure S1. The distribution of the expression values for the gene ORMDL3 in the Metabric validation set. Figure S2. The distribution of the expression values for the gene ORMDL3 in the Metabric validation set and TCGA BRCA patients. Figure S3. Scatterplots of gene expression levels vs. gene essentiality scores (GARP scores). Yellow dots are the breast cancer cells that exists in both CCLE and the shRNA screening data. The expression values and GARP scores are all adjusted by breast cancer subtypes. The purple curve is fitted by linear regression. (a) ORMDL3 (b-e) breast cancer oncogenes (f-k) breast tumor suppressors. Table S1. Multivariable survival analysis with ORMDL trimodal expression and ERBB2 expression. (DOCX 483 kb) [file 12859_2018_2582_MOESM1_ESM.docx]

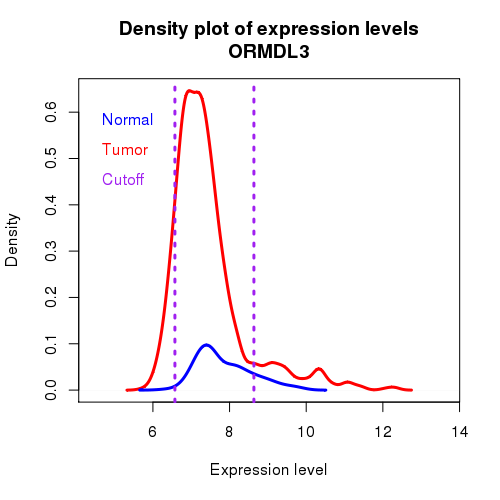


**Supplementary Fig.S1.** The distribution of the expression values for the gene ORMDL3 in the Metabric validation set.

**
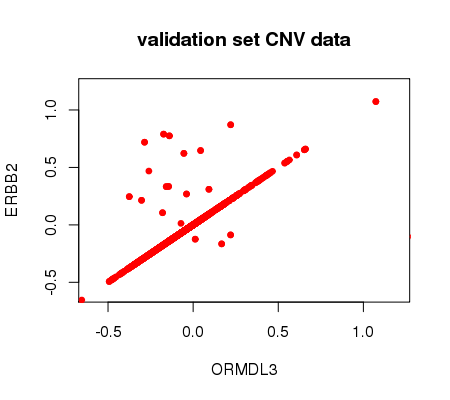

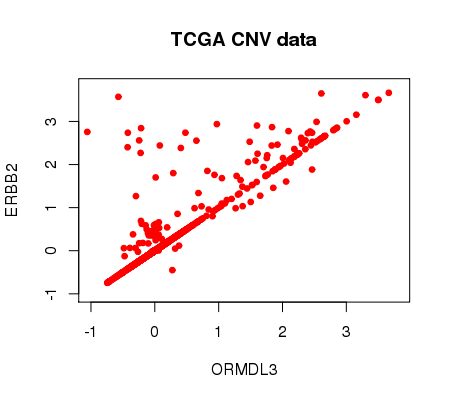
**

**(b)**

**(a)**

**Supplementary Fig.S2.** The distribution of the expression values for the gene ORMDL3 in the Metabric validation set and TCGA BRCA patients.


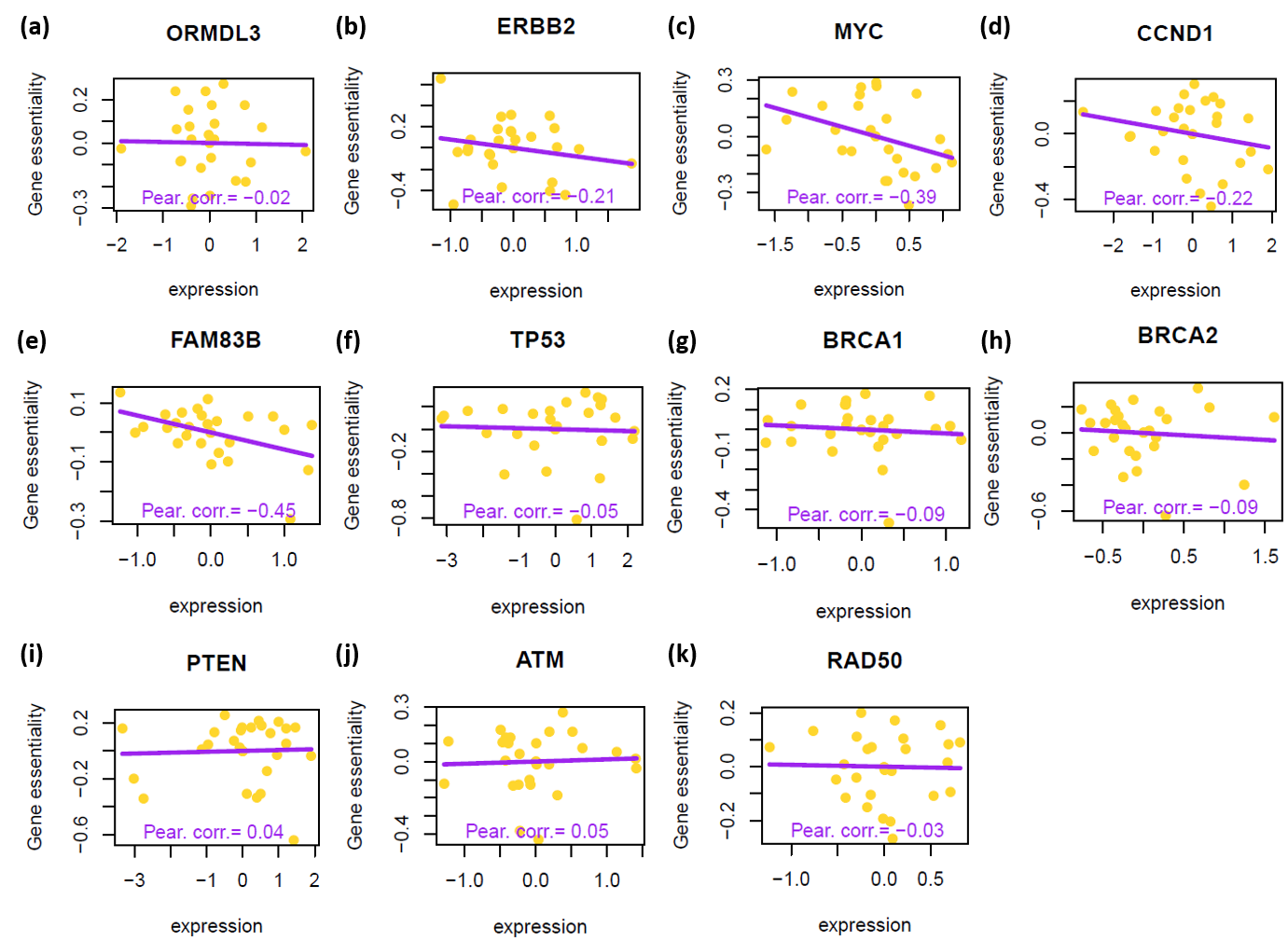


**Supplementary Fig.S3.** Scatterplots of gene expression levels vs. gene essentiality scores (GARP scores). Yellow dots are the breast cancer cells that exists in both CCLE and the shRNA screening data. The expression values and GARP scores are all adjusted by breast cancer subtypes. The purple curve is fitted by linear regression. (a) ORMDL3 (b-e) breast cancer oncogenes (f-k) breast tumor suppressors

Supplementary Table 1.  Multivariable survival analysis with ORMDL trimodal expression and ERBB2 expression

| Variables | coefficient | p-value |
| --- | --- | --- |
| ORMDL expression (“low” vs. “middle”) | 0.669 | 2.8 × 10-5 |
| ORMDL expression (“high” vs. “middle”) | 0.272 | 1.3× 10-1 |
| ERBB2 expression | 0.161 | 2.1 × 10-3 |

Analysis was done in Metabric discovery set.
